# Biotin starvation causes mitochondrial protein hyperacetylation and partial rescue by the SIRT3-like deacetylase Hst4p

**DOI:** 10.1038/ncomms8726

**Published:** 2015-07-09

**Authors:** Christian T. Madsen, Kathrine B. Sylvestersen, Clifford Young, Sara C. Larsen, Jon W. Poulsen, Marianne A. Andersen, Eva A. Palmqvist, Martin Hey-Mogensen, Per B. Jensen, Jonas T. Treebak, Michael Lisby, Michael L. Nielsen

**Affiliations:** 1 Department of Proteomics, The Novo Nordisk Foundation Center for Protein Research, Faculty of Health Sciences, University of Copenhagen, DK-2200 Copenhagen, Denmark; 2 Section of Integrative Physiology, The Novo Nordisk Foundation Center for Basic Metabolic Research, Faculty of Health Sciences, University of Copenhagen, DK-2200 Copenhagen, Denmark; 3 Department of Yeast Physiology and Cultivation, Novo Nordisk A/S, Novo Nordisk park 1, DK-2760 Måløv, Denmark; 4 Department of Obesity Biology, Novo Nordisk A/S, Novo Nordisk park 1, DK-2760 Måløv, Denmark; 5 Department of Biology, University of Copenhagen, Ole Maaloees Vej 5, DK-2200 Copenhagen, Denmark

## Abstract

The essential vitamin biotin is a covalent and tenaciously attached prosthetic group in several carboxylases that play important roles in the regulation of energy metabolism. Here we describe increased acetyl-CoA levels and mitochondrial hyperacetylation as downstream metabolic effects of biotin deficiency. Upregulated mitochondrial acetylation sites correlate with the cellular deficiency of the Hst4p deacetylase, and a biotin-starvation-induced accumulation of Hst4p in mitochondria supports a role for Hst4p in lowering mitochondrial acetylation. We show that biotin starvation and knockout of Hst4p cause alterations in cellular respiration and an increase in reactive oxygen species (ROS). These results suggest that Hst4p plays a pivotal role in biotin metabolism and cellular energy homeostasis, and supports that Hst4p is a functional yeast homologue of the sirtuin deacetylase SIRT3. With biotin deficiency being involved in various metabolic disorders, this study provides valuable insight into the metabolic effects biotin exerts on eukaryotic cells.

## Abstract (web summary)

Biotin is an essential vitamin in the regulation of energy metabolism. Here Madsen *et al.* show that biotin deficiency in yeast leads to hyperacetylation of mitochondrial proteins that is compensated for by the SIRT-like deacetylase Hst4p.

## Graphical abstract

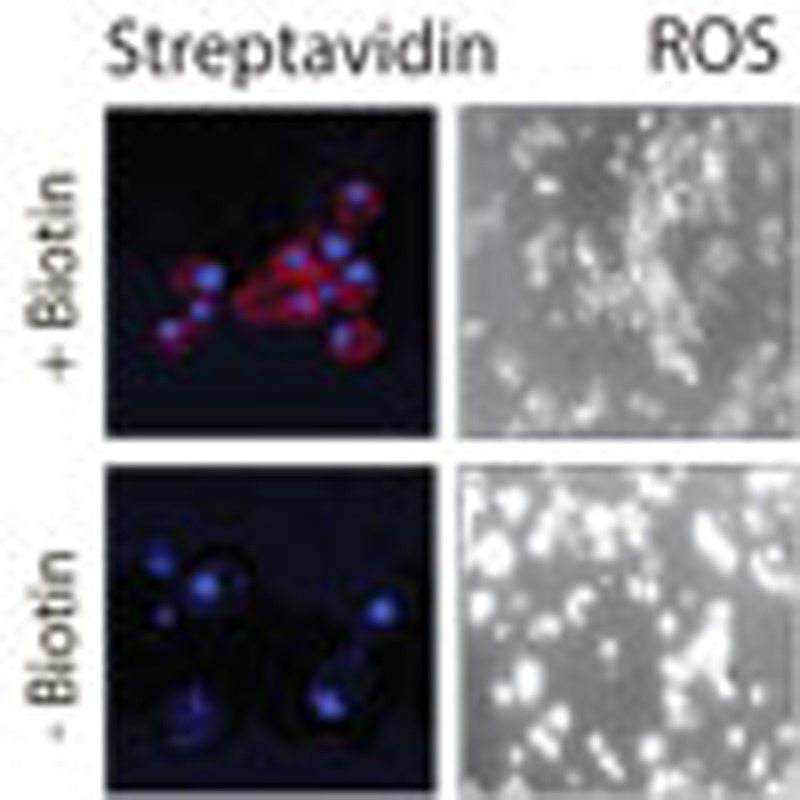

Proteins engage in a wide array of cellular events where the specificity of the interactions is largely determined by their primary amino-acid sequence. However, this can be heavily influenced by the presence of ∼200 different types of post-translational modifications (PTMs)^1^. Among these PTMs, phosphorylation, methylation, acetylation and ubiquitylation are well characterized and are also considered to be very dynamic and ubiquitous in nature^2^. Contrary to such dynamic modifications, there are some PTMs with tenacious attachment and high specificity. One such example is the essential vitamin biotin, which functions as a covalently linked prosthetic group in a handful of key metabolic enzymes^3^. These include pyruvate- and acetyl-CoA-carboxylases, which are positioned in crux points of metabolism, where they act as major regulators of acetyl-CoA flux and contribute to anaplerosis^3^.

The incorporation of biotin into carboxylases is catalysed by the essential holo-carboxylase synthetase HLCS in humans, whereas the same function is performed by the orthologue Bpl1p in yeast. The HLCS ligase is constitutively expressed in the brain and downregulated in the liver during conditions of limited biotin availability, whereas the biotinidase appears unaffected by biotin status^4^, suggesting different organs have different requirements for functional carboxylase activity^5^. The inability to metabolize and incorporate biotin into carboxylases is an autosomal recessive disorder caused by mutations in the *HLCS* gene^6^. This results in multiple carboxylase deficiencies with ketoacidosis, hyperammonemia, the excretion of abnormal organic acid metabolites and dermatitis as the most identifiable symptoms^7^. Since biotin deficiency can cause developmental defects^8^, mammals have developed a system to cope with low availability of biotin known as the biotin cycle. In this cycle a specific biotinidase enzyme scavengers biotin from biocytin, following the proteolysis of endogenous proteins. Mutations within the biotinidase gene responsible for recycling biotin are similarly problematic if not identified^9^. In contrast, *Saccharomyces cerevisiae* does not harbour any characterized biotinidase function, suggesting a more simplistic biotin utilization in unicellular eukaryotes^10^.

Although biotin is widely recognized to be involved in several biological processes, the molecular details surrounding its exact cellular function remain elusive. While the coenzymatic role of biotin in carboxyl-group transfer has been well established, much less is known about the metabolic consequences of biotin deprivation at the cellular level. This is further illustrated by mouse studies demonstrating biotin greatly improves glucose metabolism and insulin sensitivity^11^. Moreover, it is widely recognized that biotin functions as a regulator of gene expression in both prokaryotic and eukaryotic systems^12^,^13^,^14^. However, the underlying mechanisms still remains elusive.

It has recently been shown that cellular acetyl-CoA levels affect protein acetylation in a non-enzymatic manner^15^. We therefore hypothesized that alterations in biotin levels would perturb the acetyl-CoA flux through the biotin-dependent carboxylases, and in turn affect downstream acetylation events. To investigate this in more detail, we selected *S. cerevisiae* as a model organism since the internal biotin pool can be directly manipulated from the growth media^16^.

In the present work, we provide new data for a better understanding of the integrated *in vivo* biotin physiology in a eukaryotic cell. To this end, we make use of recent advances in proteomics technologies, such as high-accuracy quantitative mass spectrometry (MS), to dissect the functional consequences of biotin starvation on the proteome, acetylome and metabolome levels (Fig. 1a). Our results demonstrate that biotin deficiency manifests itself into mitochondrial hyperacetylation, and our large-scale proteomic data revealed that upregulated mitochondrial acetylation sites correlate with the cellular deficiency of the Hst4p deacetylase. Studying the cellular localization of Hst4p by immunofluorescence revealed a biotin-dependent accumulation of Hst4p in the mitochondria, which supports a role for Hst4p in lowering mitochondrial acetylation. We show that biotin starvation and knockout of Hst4p impairs metabolic flexibility through alterations in cellular respiration and an increase in reactive oxygen species (ROS). These results consolidate Hst4p as an important link between biotin metabolism and cellular energy homeostasis, and supports that Hst4p is a functional yeast homologue of the mammalian sirtuin deacetylase SIRT3.

## Results

### Biotinylation is dynamically controlled by availability

**Figure 1:**
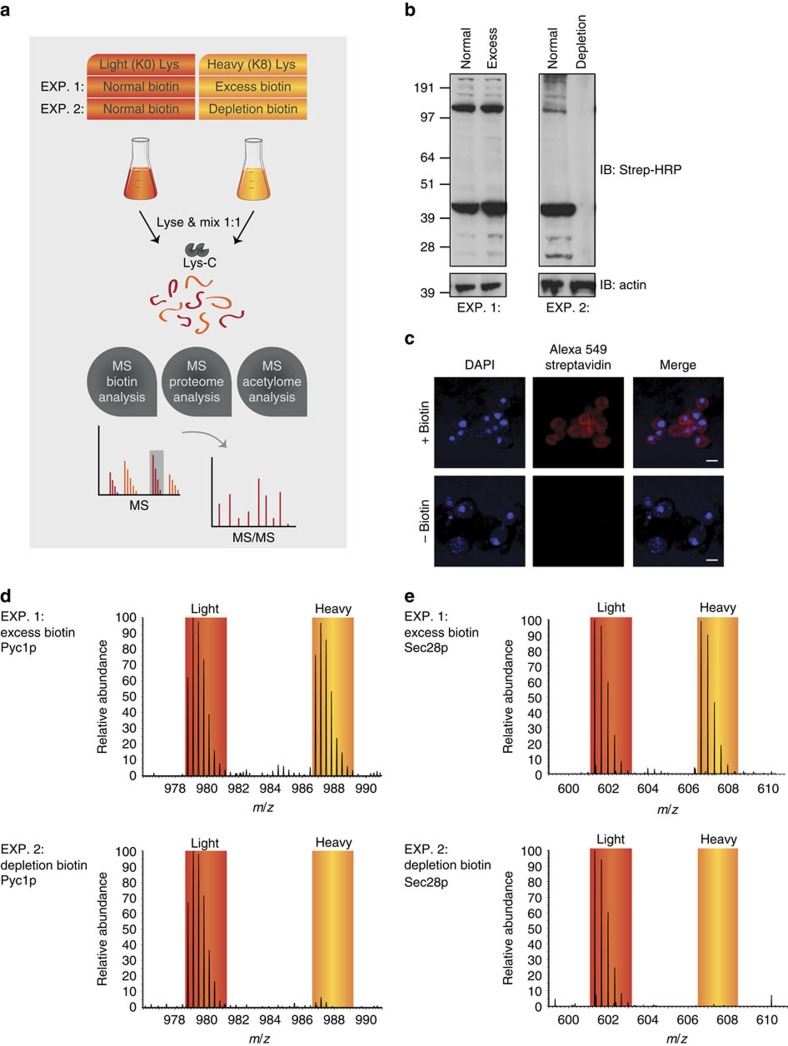
Protein biotinylation is dynamically controlled by availability. (**a**) Quantitative SILAC strategy to determine the effect of biotin excess or biotin depletion. Experiment 1: WT yeast grown with 2 μg l^−1^ biotin (normal) in light-SILAC media and compared with WT yeast grown with 200 μg l^−1^ biotin (excess) in heavy SILAC. Experiment 2: similar as above but with biotin depletion in the ‘heavy'-SILAC state. Each SILAC mix is used for biotin enrichment, proteome- and acetylome analysis by liquid chromatography–MS/MS. (**b**) Streptavidin-HRP western blot analysis of the biotinylated protein content from each cellular state as depicted in **a**. (**c**) Immunofluorescence microscopy of protein-bound and free biotin content from experiment 2 using the Alexa 549 streptavidin antibody. Nucleus is stained with 4,6-diamidino-2-phenylindole (DAPI). Scale bar, 5 μm. (**d**) MS spectra of the relative abundance of specific Pyc1p- and Sec28p-biotinylated peptide from excess (experiment 1). (**e**) Similar biotinylated peptide analysis performed in depletion (experiment 2).

To measure the cellular response to biotin availability, we metabolically labelled all proteins using stable isotope labelling of amino acids in cell culture (SILAC)^17^. Using this set-up we determined the cellular response of biotin, proteome and acetylome in cells exposed to normal amounts of biotin against those exposed to 100-fold excess (experiment 1), or complete depletion (experiment 2) by quantitative MS (Fig. 1a). Since biotinylated proteins have different half-lives (Supplementary Fig. 1a)^18^, complete biotin depletion was achieved by two sequential overnight dilutions (Supplementary Fig. 1b). Western blot analysis confirmed the cellular response to an excess amount of biotin, as no changes in the overall biotinylation pattern was observed (Fig. 1b), while the extent of biotinylated proteins in biotin-depleted cells was considerably lower (Fig. 1b). Fluorescence microscopy analyses further confirmed that biotin was widely distributed across all cellular compartments under normal growth conditions, whereas the fluorescent biotin signal was undetectable under biotin starvation conditions (Fig. 1c). However, western blot and microscopy analyses only measure the total cellular state of the biotin, and are thus inadequate to determine the extent of biotinylation at a molecular level. Thus, to investigate the extent of biotinylation at the molecular level using MS, we employed a streptavidin-based peptide enrichment method^19^ for the biotin-depleted SILAC samples (Supplementary Fig. 1c). This analysis revealed the site-specific lysine biotinylation extent within individual proteins, and confirmed that exposing cells to excess amount of biotin does not appreciably influence the cellular lysine biotinylation (Fig. 1d). In contrast, biotinylated proteins were completely diminished in their lysine biotinylation occupancy upon biotin starvation (Fig. 1e). Our analysis confirmed the known biotin-dependent carboxylases, while several novel biotinylated proteins (Supplementary Data 1; Supplementary Fig. 1d,e) were discovered and their identification confirmed using biochemical methods (Supplementary Fig. 1f,g). Collectively, these results confirm that the experimental conditions for studying the cellular biotin response of yeast are appropriate.

### Biotin depletion causes a retrograde-like signalling response

**Figure 2:**
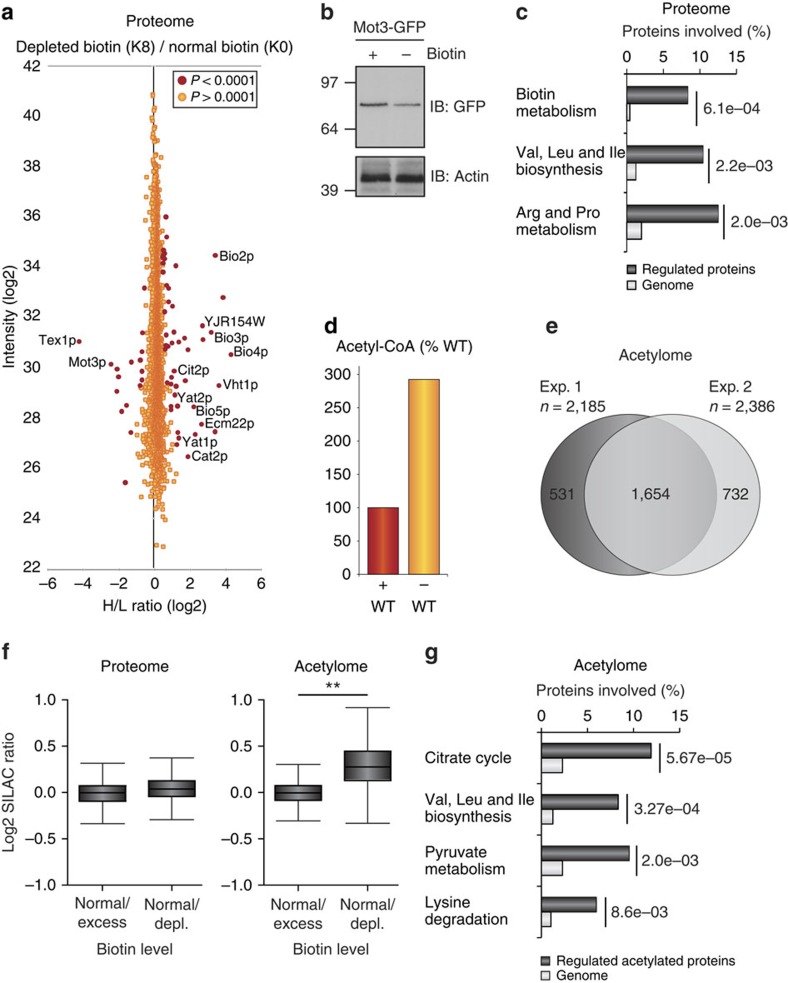
Biotin starvation induces a retrograde-like proteome response and mitochondrial hyperacetylation. (**a**) Proteome perturbations in response to biotin depletion. Signal intensities of identified proteins plotted against the corresponding normalized heavy/light ratio. Significant outliers in red (false discovery rate of *P*<0.0001) was determined using a Benjamini–Hochberg correction for multiple hypothesis testing. (**b**) Western blot of Mot3–GFP grown in the presence or absence of biotin and blotted with a GFP antibody. (**c**) Gene ontology annotation analyses of regulated proteins compared with their respective genome abundance. *P* values calculated separately for each GO term using Fisher's exact test. (**d**) Relative biotin-dependent acetyl-CoA levels of WT yeast cells. (**e**) Venn diagram showing the overlap of quantified acetylation sites in the light-SILAC state from two independent experiments with either excess or depletion of biotin. (**f**) Box-plot analysis comparing the distribution of quantified proteins at the proteome and acetylome level of representative biotin excess or depletion experiments. The line across the box identifies the median sample value, the ends of the box are the 25th and 75th percentiles and whiskers represent minimum and maximum values. An unpaired *t*-test was performed to test difference between the mean log2 SILAC ratios. **P<0.01 (**g**) GO annotation of regulated acetylated proteins compared with their respective genome abundance from the biotin-depletion experiment. *P* values calculated separately for each GO term using Fisher's exact test.

We first determined the proteome expression changes associated with excess and depleted amounts of biotin using a quantitative SILAC strategy as previously described^17^. In this comparison, we quantified the protein expression of >3,000 yeast proteins in response to biotin availability (Supplementary Data 2). In agreement with western blot analysis (Fig. 1b), an excess amount of biotin did not appreciably change the overall proteome expression level (Supplementary Fig. 2a; Supplementary Data 2), suggesting a controlled cellular uptake and metabolism of biotin. In contrast, yeast cells depleted of biotin exhibited a strong cellular response (Fig. 2a), including significant upregulation of all proteins involved in biotin metabolism (Bio3p, Bio4p, Bio2p, Bio5p, Vht1p, Vhr1p and Bpl1p) (Supplementary Data 2). In addition, the biotin proteome data revealed altered protein expression of the two ergosterol transcription factors Ecm22p and Mot3p. Notably, the expression level of the mammalian Ecm22p orthologue (SREPB1) has been described as being biotin dependent^20^, and we additionally confirmed the biotin-dependent downregulation of Mot3p by western blot analysis (Fig. 2b). These transcription factors are regulated in a complex manner dependent on oxygen^21^ and suggests that biotin availability affects oxygen consumption^22^.

Another proteome response to biotin starvation included upregulation of proteins involved in branched chain amino-acid (BCAA) synthesis (Fig. 2c), in line with organic aciduria being a metabolic manifestation of biotin deficiency^23^. With changes in BCAA flux directly coupled to the tricarboxylic acid (TCA) cycle through the metabolites acetyl-CoA and succinyl-CoA, these results imply that increased BCAA metabolism influences the cellular distribution of acetyl-CoA^24^. This furthered by the observation of several proteins responsible for the shuttling of acetyl-CoA into mitochondria were increased in their protein expression, including members of the acetyl-carnitine shuttle (Yat1p, Yat2p and Cat2p) and peroxisomal Cit2p (Supplementary Data 2)^25^. Moreover, activation of the ornithine/arginine pathway (Arg3p, Arg5p and Arg8p) can lead to an upregulation of nitrogen disposal during biotin starvation. Together, the proteome data suggest upregulation of metabolic processes involved in the retrograde signalling response, where metabolic pathways are recast as a response to a dysfunctional mitochondria^26^. It is worth noting that the regulated proteome data follow previously reported data on gene transcript changes in biotin-starved yeast^13^. To confirm that biotin starvation indeed causes a substantial increase in cellular acetyl-CoA levels, we compared acetyl-CoA levels in wild-type (WT) cells grown in biotin-fed and -starvation conditions (Fig. 2d)^27^. The increased acetyl-CoA levels could be a strong indicator of increased levels in lysine acetylation^15^.

### Biotin starvation affects mitochondrial protein acetylation

To establish whether biotin availability causes a regulatory effect on protein acetylation, a MS-based acetyl-peptide immunoprecipitation enrichment procedure was performed^28^. From two SILAC experiments representing normal/excess (experiment 1) and normal/depleted amounts of biotin (experiment 2), respectively, we quantified >2,900 acetylation sites (Fig. 2e; Supplementary Data 3). Approximately 75% of the acetylation sites were identified in both SILAC experiments (Fig. 2e), signifying high reproducibility in the experimental set-up (*P*<1e−200) and further supported with a Pearson correlation of 0.95 between biological replicate analyses (Supplementary Fig. 2b). Similar to protein expression levels, our acetylome analysis revealed no discernable difference between cells exposed to normal and excess amounts of biotin (experiment 1, Supplementary Fig. 2c; Supplementary Data 3), whereas biotin depletion (experiment 2) caused a broader effect on the cellular acetylation distribution (Fig. 2f). A Gene Ontology (GO) enrichment analysis revealed that the affected acetylation sites primarily reside on mitochondrial proteins (Supplementary Fig. 2d). Specifically, the acetylated proteins were tightly associated with the TCA cycle, BCAA synthesis, pyruvate metabolism and lysine-degradation pathways, revealing that biotin starvation has substantial downstream metabolic effects (Fig. 2g).

To assess the overall levels of lysine acetylation by a complementary method, we performed western blot analysis of mitochondrial proteins^29^ (Supplementary Fig. 3). These data corroborate that biotin starvation causes minor changes in lysine acetylation content, in agreement with our quantitative proteomics data. Since BCAA flux is coupled to both acetyl-CoA and succinyl-CoA, we performed a similar western blot analysis for mitochondrial lysine succinylation (Supplementary Fig. 3), revealing that the mitochondrial abundance of lysine succinylation is lower compared with lysine acetylation. This is not surprising considering that overall cellular levels of succinyl-CoA are significantly lower than acetyl-CoA^30^ and that succinyl-CoA is an intermediate, while acetyl-CoA is the major carbon precursor of the TCA.

### Mitochondrial hyperacetylation is counteracted by Hst4p

**Figure 3:**
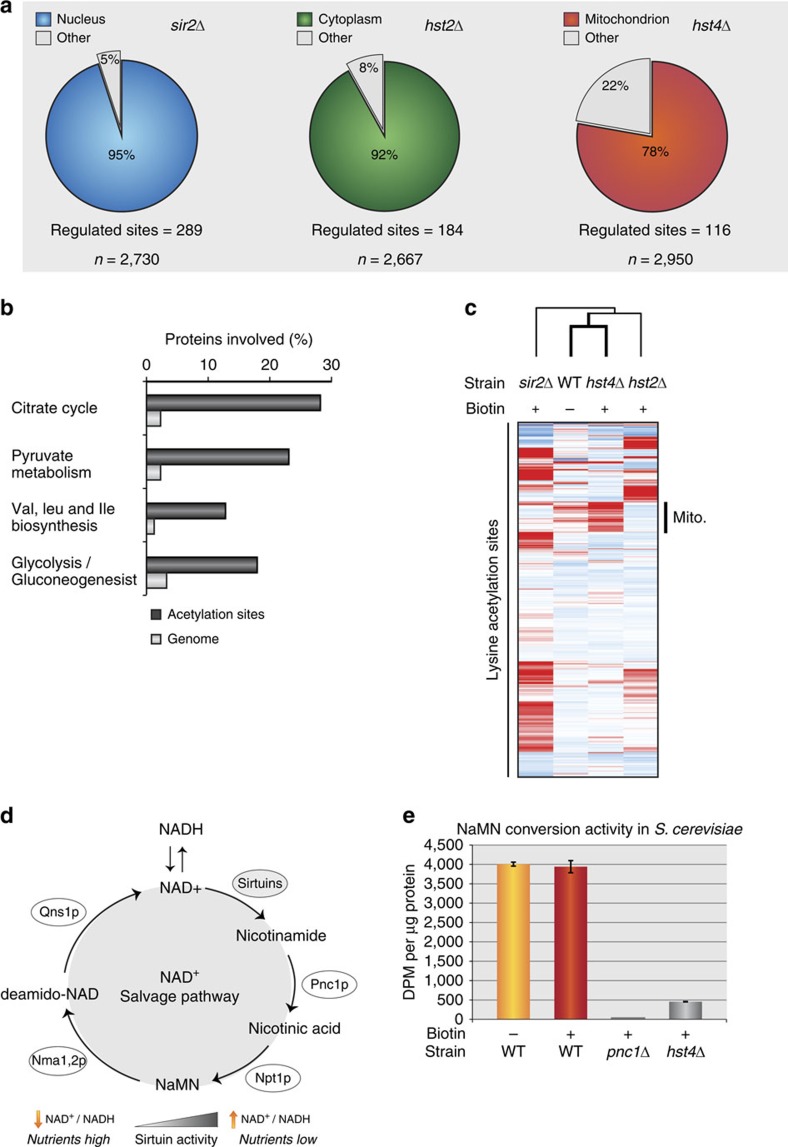
Mitochondrial acetylation overlaps at a site-specific level between an *hst4*Δ mutant and a biotin-starved WT. (**a**) The cellular distribution of regulated acetylation sites for sirtuin deacetylases Sir2p, Hst2p and Hst4p. Total number of quantified sites are shown below. (**b**) GO annotation of regulated proteins compared with their respective genome abundance from the *hst4*Δ acetylation experiment. (**c**) Hierarchical cluster analysis of commonly identified lysine acetylation sites in sirtuin mutants and biotin-starved WT. (**d**) The NAD^+^ salvage pathway. NAD^+^ is converted to nicotinamide during high level of sirtuin deacetylase activity at nutrient-low conditions. Through enzymatic activities of Pnc1p and Npt1p, nicotinamide is converted to nicotinic acid mononucleotide (NaMN). (**e**) The flux through the NAD^+^ salvage pathway in WT cells with or without biotin compared with *pnc1*Δ and *hst4*Δ mutant strains. [^14^C]-nicotinamide conversion to NaMN measured by scintillation normalized to the amount of protein. Error bars indicate s.d. in three biological replica experiments.

Yeast expresses several NAD^+^-dependent sirtuin deacetylases with similarity to the mitochondrial confined SIRT3 in mammals^31^. To investigate whether a yeast sirtuin could be associated with the observed mitochondrial hyperacetylation, we mapped the acetylome of several deacetylase mutants (*sir2*Δ, *hst2*Δ and *hst4*Δ). More than 72% of the acetylation sites identified in the mutants were also present in the biotin-depletion acetylome (Supplementary Fig. 4a). However, the altered acetylation sites differed notably between deletion strains, with distinct differences in their target substrates and cellular localization (Supplementary Data 4). In the *sir2*Δ mutant, 95% of affected acetylation sites were exclusively localized to the nucleus, while 92% of altered acetylation sites in the *hst2*Δ mutant were cytoplasmic proteins (Fig. 3a; Supplementary Data 4). This is in agreement with the known cellular roles of Sir2p and Hst2p deacetylases^32^,^33^, and confirms that the acetylation enrichment procedure targets substrates that co-localize with the investigated sirtuin^34^. Conversely, 78% of affected acetylation sites in the *hst4*Δ mutant reside exclusively on mitochondrial proteins (Fig. 3a; Supplementary Data 4). GO analysis revealed that the Hst4p-altered acetylated proteins belong to the same biological processes affected upon biotin starvation (TCA cycle, BCAA and pyruvate metabolism) (Fig. 3b). To investigate this overlap in more detail, a hierarchical cluster analysis of all acetylation sites commonly identified in all of the acetylome experiments (comparing biotin depletion with *sir2*Δ, *hst2*Δ and *hst4*Δ) was performed. The biotin-depleted WT acetylome clustered together with the *hst4*Δ acetylome, with both strains exhibiting a significant upregulation of the same mitochondrial lysine acetylation sites (Fig. 3c). Comparison of the commonly altered lysine acetylation sites revealed that almost every protein in the TCA cycle is an enzymatic target for both Hst4p and biotin depletion (Supplementary Fig. 4b). Considering that Hst4p is a lysine deacetylase, these results suggest that Hst4p may attempt to clear mitochondrial acetylation events triggered by biotin starvation. If Hst4p participates in dispersing mitochondrial hyperacetylation, a link between Hst4p activity and the NAD^+^ salvage pathway would be expected as previously described^35^. Since NAD^+^ is the substrate of sirtuins during the deacetylation reaction, the NAD^+^ dependence of these enzymes provides a link between metabolism and the cellular redox state^36^,^37^. Thus, to investigate this in more detail we measured the flux through the NAD^+^ salvage pathway for WT and *hst4*Δ cells^38^ (Fig. 3d). Here we found that biotin availability did not cause any changes through the NAD^+^ salvage pathway in a WT genetic background (Fig. 3e). In contrast, a *hst4*Δ strain exhibited severely reduced NAD^+^ conversion capabilities linking Hst4p to mitochondrial nicotinamide regeneration^39^, and supporting that Hst4p could be involved in rescuing mitochondrial hyperacetylation elicited by biotin starvation. Notably, the severely reduced potential to regenerate NAD^+^ in *hst4*Δ cells was similar to the known effects of a *pnc1*Δ strain, where Pnc1p is an enzyme linked to mitochondrial NAD^+^ regeneration (Fig. 3e)^40^.

### Hst4p accumulates in mitochondria during biotin starvation

**Figure 4:**
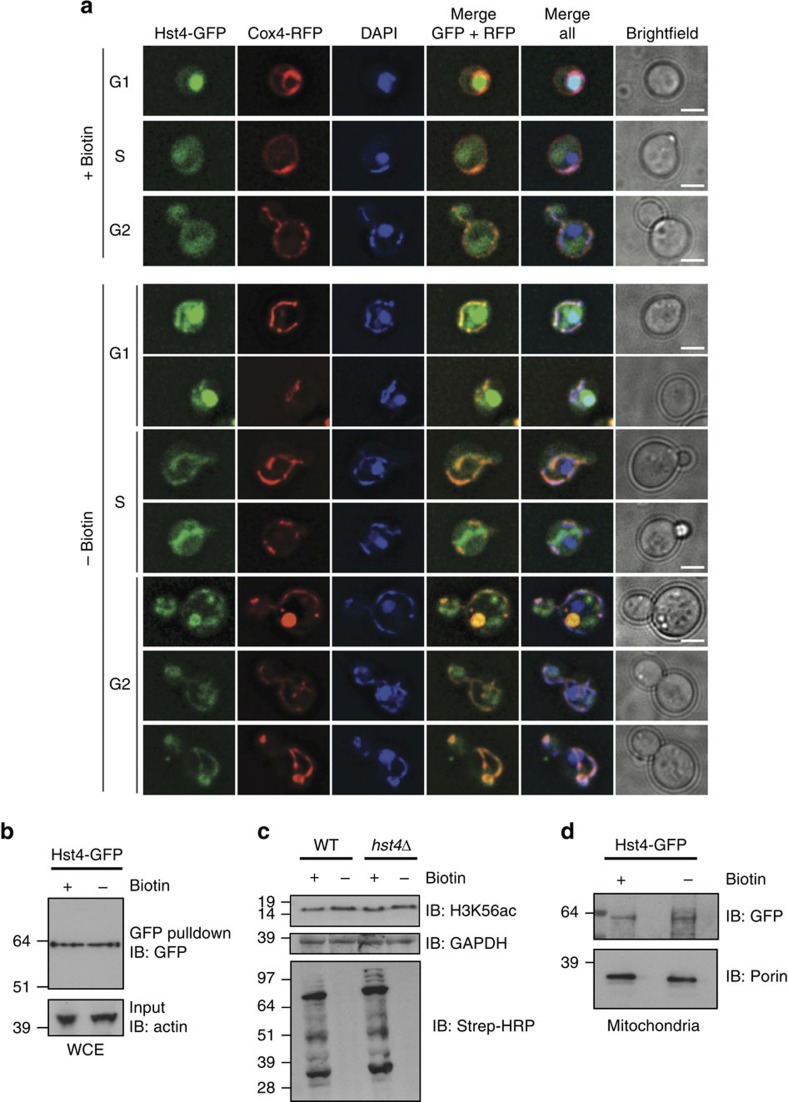
Mitochondrial Hst4p localization depends on biotin, and H3K56 acetylation is unaffected in the *hst4*Δ mutant or by biotin availability. (**a**) Biotin and cell-cycle-dependent localization of Hst4–GFP and mitochondrial marker Cox4–RFP in G1, S and G2. Biotin starvation led to a marked accumulation of Hst4–GFP in the mitochondria evidenced by co-localization with Cox4 and 4,6-diamidino-2-phenylindole (DAPI) staining. Scale bar, 2 μm. (**b**) Hst4–GFP expression dependent on biotin availability shown by western blot analysis on whole-cell extracts. (**c**) H3K56 acetylation is unaffected by biotin availability in both WT and *hst4*Δ genetic background, as evidenced by western blot analysis. Control blots for complete biotin depletion was performed. (**d**) Hst4–GFP expression in mitochondrial fractions on biotin availability. Control blot for mitochondrial fraction using Porin specific antibody. See Supplementary Fig. 3 for additional controls.

To support our observations that Hst4p may participate in mitochondrial deacetylation, we investigated the cell cycle and biotin-dependent localization of Hst4p by fluorescence microscopy. To this end, the mitochondrial marker Cox4 tagged with red fluorescent protein (Cox4–RFP) was introduced into a strain expressing Hst4–GFP from its endogenous promoter^41^. Irrespective of the presence of biotin, Hst4–GFP exhibited a cell-cycle-dependent exclusion from the nucleus during the S/G2 phase (budded cells) of the cell cycle, consistent with its role in histone deacetylation after DNA replication (Fig. 4a)^42^. Hst4–GFP was present in the cytoplasm and in mitochondria at all phases of the cell cycle, as evidenced by the co-localization of Cox4–RFP and 4,6-diamidino-2-phenylindole staining. Considering that Cox4 is located on the matrix side of the inner mitochondrial membrane, the observed co-localization confirms that Hst4p localizes to the same mitochondrial region. However, biotin starvation led to a marked accumulation of Hst4–GFP in mitochondria (Fig. 4a), consistent with our observed changes in mitochondrial acetylation. Hst4–GFP showed no discernable difference in expression in response to biotin starvation (Fig. 4b), demonstrating that mitochondrial accumulation was a relocalization event.

It has previously been demonstrated that Hst4p plays a redundant role in histone H3K56 deacetylation after the completion of DNA replication^43^. However, the effect of Hst4p on H3K56 acetylation was only observed with simultaneous removal of Hst3p^43^. Hence, to investigate whether Hst4p may affect mitochondrial protein acetylation through indirect regulation of H3K56 acetylation, as previously demonstrated for SIRT7 in mammals^44^, we investigated the expression levels of H3K56 acetylation during biotin depletion (Fig. 4c). We observed no biotin-dependent changes of H3K56 acetylation, and with the substantial overlap of regulated acetylation sites between *hst4*Δ and biotin depletion, our data support that Hst4p is directly involved in rescuing the metabolic consequences of biotin starvation.

Next, we performed an enrichment of mitochondrial proteins in a Hst4–GFP strain following a standard procedure^29^. Using a specific green fluorescent protein (GFP) antibody on the mitochondrial fraction, we could confirm the presence of Hst4p (Fig. 4d). Notably, the mitochondrial presence of Hst4p in cells supplemented with biotin follows our observation in Fig. 3a, which shows upregulation of mitochondrial acetylation sites in *hst4*Δ. Hence, these data corroborate that Hst4p is localized to mitochondria in yeast (Fig. 4d), and our microscopy analysis show that Hst4p is partially localized to mitochondria in a cell-cycle-dependent manner (Fig. 4a).

### Hst4p protects the mitochondria from biotin starvation

**Figure 5:**
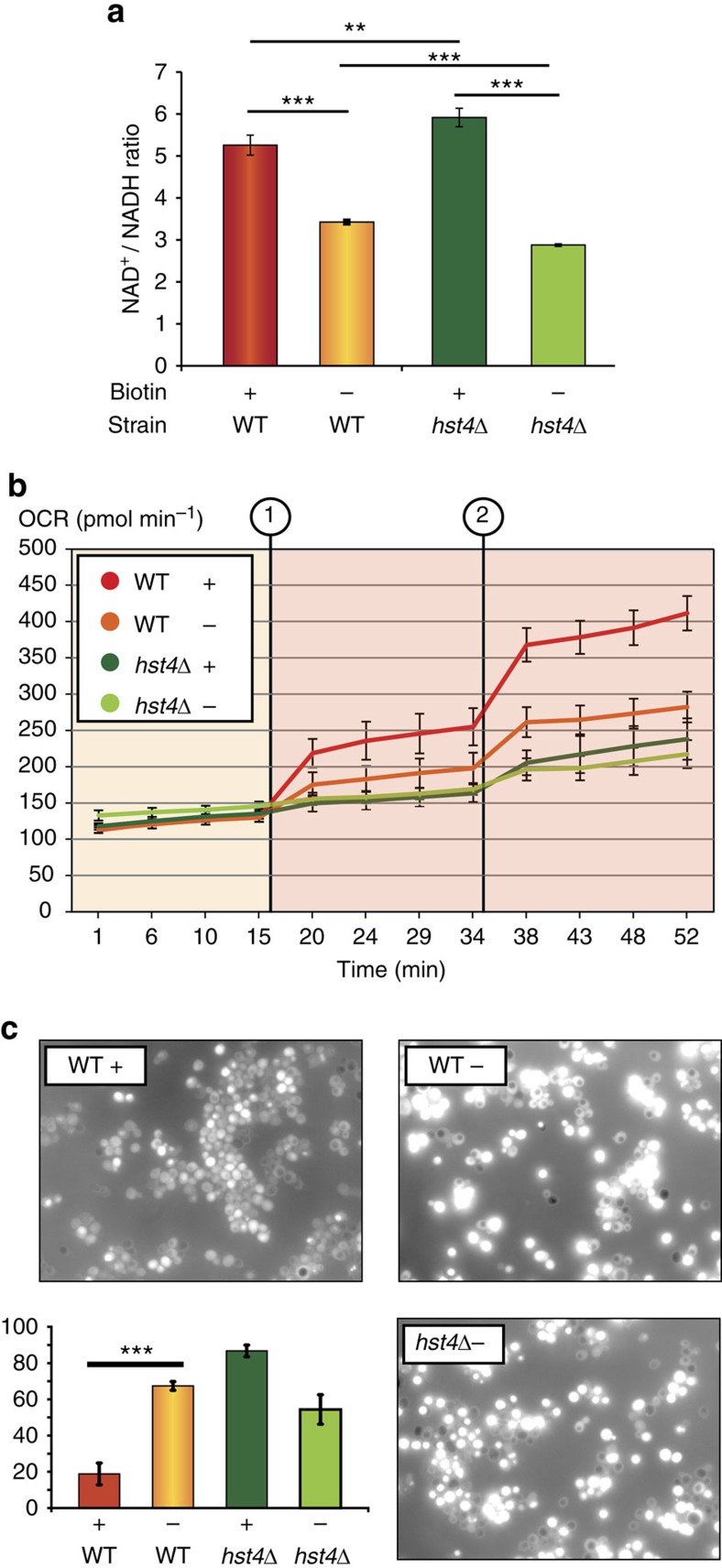
Mitochondrial consequences of biotin deprivation. (**a**) Fluorescent measurements of NAD^+^ and NADH in WT and the *hst4*Δ mutant, as quantified from standard curve measurements. An unpaired *t*-test was performed to test the difference between the mean from three biological replica experiments. (**b**) Basal respiration and maximum respiratory capacity measured for WT and the *hst4*Δ mutant in *n*=12 technical replica by the oxygen consumption rate (OCR). Error bars represent s.d. Decoupling of oxidative phosphorylation at time point 1 with 1.5 μM FCCP and at time point 2 with 3 μM FCCP as final concentration. (**c**) Representative pictures of H_2_DCF-DA-stained cells. Scale bar, 25 μm. The bar diagram shows percentage of fluorescent cells compared with non-fluorescent cells as mean value±s.d. from quadruplicate counting of *n*>100 cells. **P<0.01, ***P<0.001.

To investigate the mitochondrial consequences of biotin starvation, we decided to measure the biotin-dependent NAD^+^/NADH ratio for WT and *hst4*Δ cells. During biotin starvation, WT cells exhibited a significant decrease in the NAD^+^/NADH ratio (Fig. 5a; Supplementary Fig. 5a), which is in agreement with reports describing the cellular consequences of mitochondrial hyperacetylation^45^. In contrast, a small increase in NAD^+^/NADH ratio was observed for *hst4*Δ cells when biotin was readily available (Fig. 5a), which is analogous to the deletion of other NAD salvage pathway components^46^. Cells deprived for both biotin and Hst4p exhibited a significantly reduced NAD^+^/NADH ratio compared with WT cells deprived of biotin. However, only *hst4*Δ cells supplemented with biotin exhibited a significant increase in the level of acetyl-CoA compared with its WT counterpart (Supplementary Fig. 5b), confirming that Hst4p indeed has mitochondrial and metabolic obligations.

As several metabolic mechanisms may contribute to the redox state of yeast cells, such as decreased anaplerosis as a consequence of pyruvate carboxylase deficiency^12^, the measured NAD^+^/NADH values represent a global cellular ratio. Hence, to investigate the mitochondrial consequences of *hst4*Δ and biotin deficiency, we measured the oxygen consumption rate using a SeaHorse XF analyzer to ascertain respiration efficiency (Fig. 5b). Basal respiration was largely unaffected for WT and *hst4*Δ strains, independent of biotin availability, hereby supporting our previous cellular NAD^+^/NADH measurements for *hst4*Δ cells. In contrast, when the maximum respiratory capacity was tested by decoupling oxidative phosphorylation^47^, a significant decrease in respiration efficiency in WT cells lacking biotin was observed. Under mitochondrial stress conditions, the biotin-starved WT cells mimicked the respiratory efficiency of *hst4*Δ cells (Fig. 5b). Notably, these results were reproduced in cell suspension (Supplementary Fig. 5c–e), which confirm that biotin starvation causes mitochondrial dysfunction and that Hst4p notably affects mitochondria under stress conditions.

Ineffective mitochondrial respiration can result in excessive ROS production^48^. Since we observed increased acetylation of superoxide dismutase (Sod2p) (Supplementary Data 4) and that the acetylated mammalian homologue is known to have a lower capacity to remove ROS^49^, we decided to investigate the mitochondrial consequences of biotin scarcity. ROS levels were significantly increased for both biotin-starved WT cells (Fig. 5c) and the *hst4*Δ mutant supplemented with biotin, where the latter situation is most likely due to the acetylation-dependent inactivation of Sod1p and Sod2p caused by the lack of Hst4p deacetylase activity (Supplementary Data 4). Collectively, these results confirm that Hst4p dampens mitochondrial acetylation events in the respiratory chain and prevents ROS formation. Moreover, the relatively reduced ROS production in the *hst4*Δ mutant starved for biotin (Fig. 5c) follows the lowered NAD^+^/NADH ratio under these conditions (Fig. 5a), highlighting the importance of NAD^+^-dependent mitochondrial ROS generation^50^.

## Discussion

Our results demonstrate that reorientation of the nucleocytosolic acetyl-CoA pool during biotin scarcity manifests itself into mitochondrial hyperacetylation, and causes wide-spread downstream effects upon cellular respiration and redox balance. The observed responses are reminiscent of mammalian cells lacking the mitochondrial protein SIRT3 (ref. 51), consistent with a rat study demonstrating that biotin deprivation is inversely related to cellular energy status, resulting in increased insulin sensitivity^52^. Moreover, oxidative stress contributes to the progression of various human diseases such as type 2 diabetes, where an increased supply of energy substrates results in excessive mitochondrial ROS^53^. As a result, our presented data entail a valuable resource that details the link between biotin starvation and ROS production in eukaryotic cells, and delineates the effects of biotin availability upon insulin sensitivity through acetyl-CoA flux and mitochondrial hyperacetylation.

Although the vast majority of lysine acetylation in yeast occurs at low levels^15^, the basal level of acetylation is higher in mitochondria compared with other compartments^15^. Indicative of a higher concentration of acetyl-CoA in this organelle and therefore the organelle most likely affected by biotin depletion as demonstrated by our results.

It should be noted that low stoichiometry should not be viewed as an indicator for acetylation being biologically irrelevant, as acetylation may be confined only to a subset of the cellular protein pool, with such subset-specific modifications being masked using proteome-wide analysis as described here. Although the overall acetylation levels might be low and may happen through non-enzymatic reactions as compared with other PTMs, the role of mitochondrial lysine acetylation in regulating diverse metabolic pathways and its relevance to the stress response is well described^54^,^55^, demonstrating the biological importance of mitochondrial acetylation, which is further supported by the mitochondrial consequences caused by biotin depletion described here.

Other metabolites may be affected by biotin depletion as well, but presumably not as a direct effect of biotin availability. Although succinyl-CoA is tightly associated with BCAA and an important intermediate in the TCA, the levels of succinyl-CoA are not directly associated with the biotin-dependent carboxylases contrary to acetyl-CoA. Particularly in yeast which lacks the biotin-dependent propionyl-CoA carboxylases, which in mammals generates (S)-methylmalonyl-CoA that can be converted into succinyl-CoA by methylmalonyl-CoA mutase. Moreover, biotin starvation causes reduced ATP production by the TCA, which in turn activates the energy stress sensor AMP kinase in mammals^12^. Hence, biotin starvation may additionally cause downstream cellular changes related to protein phosphorylation, but not as a direct cause of biotin availability (carboxylase inactivation). Investigations of other PTMs, such as lysine succinylation and protein phosphorylation, may indeed reveal novel insights into the downstream effects of biotin depletion. However, such analyses are beyond the scope of this study.

We find that biotin depletion involves actions of the sirtuins, a set of deacetylases shown to improve glucose metabolism. In particular, we describe that Hst4p is localized to mitochondria upon biotin scarcity and exhibits SIRT3-like activities including the deacetylation of mitochondrial substrates^34^, cellular respiration^56^ and ROS levels^57^. However, many of these consequences are only fully manifested upon mitochondrial relocation, as observed during biotin starvation. Still, our results provide evidence that Hst4p plays a functional role in yeast similar to SIRT3 in mammals.

SIRT3 functions as a tumour suppressor^58^, and mice lacking SIRT3 are more susceptible to developing cancer. Whether biotin availability plays a similar role in cancer remains to be investigated. However, a correlation between biotin intake and the expression of oncogenes in small-cell lung cancer has previously been described^59^, and the biotinidase has been suggested as a potential serological biomarker for the detection of breast cancer^60^.

Besides its role in metabolism, biotin is widely recognized as a regulator of gene expression in both prokaryotic and eukaryotic systems^12^. A mitochondrial acetyl-CoA reorientation during biotin starvation might also affect the nuclear acetyl-CoA pool and subsequently the epigenetic landscape of histone acetylation, although our acetylation analysis did not reveal any significant regulation of nuclear protein acetylation during biotin starvation. Thus, to decipher the long-term consequences of biotin deficiency would require elaborate investigations into the genetic and nuclear effects of biotin deficiency.

In conclusion, the presented study describes novel aspects of vitamin-dependent metabolism and demonstrates how cells deprived of a single coenzyme cause multiple downstream effects on the proteome, acetylome and metabolome. Our data therefore contribute to a better understanding of the complex *in vivo* biotin physiology in eukaryotes.

## Methods

### Yeast growth condition

*S. cerevisiae* knockout strains were purchased from Thermo Scientific Open Biosystems. The parental WT genetic background is BY4742 (*MATα his3Δ1 leu2Δ0 lys2Δ0 ura3Δ0*). GFP-tagged yeast strains (*MAT***a** *his3▵1 leu2▵0 met15▵0 ura3▵0 yfg-GFP::HIS3*) are described elsewhere^41^. For SILAC experiments, yeast cells were grown in synthetic complete media (0.67% YNB devoid of biotin, Bio 101 Inc., 2% glucose and 0.2% dropout mix from USBiological), supplemented where indicated with biotin (Sigma-Aldrich) to 2 μg l^−1^ (normal conditions) or 200 μg l^−1^ (excess conditions) and either regular lysine (light-SILAC condition) or a stable isotope of 13C6-15N2-labelled lysine (heavy-SILAC condition) as previously described^17^.

### Site-directed mutagenesis

The s*ec28* K27R yeast mutant was engineered by the *delitto perfetto* mutagenesis^61^. Two PCR products containing the *Kluyveromyces* *lactis URA3* (KlURA3) marker were amplified as overlapping 5′ and 3′ fragments from plasmids pWJ1164 and pWJ1165 using primers TOFT-321/382 and TOFT-325/385, respectively. The KlURA3 marker fragments were extended by fusion PCR with regions of homology to the *sec28* loci using TOFT-383/384 and TOFT-386/387, and transformed simultaneously into both a *sec28* wt and *sec28-GFP* genetic background using standard methods^62^. Integration events were selected for on SC-URA plates and confirmed by PCR (TOFT-321/388) to be in the *sec28* loci. A second fusion PCR product containing the K27R alteration (TOFT-389/390 and TOFT-391/392) was subsequently transformed into the above strain and selected for on 5-fluorouracil plates. Correct clones were confirmed by PCR (TOFT-383/393) and sequencing. All primer sequences can be found in Supplementary Data 5.

### GFP-pull-down experiments and western blotting

Fifty microlitre of GFP-Trap A agarose beads (Chromotek) was washed in 500 μl buffer (100 mM Tris-HCl, pH 8.0, 150 mM NaCl, 1 mM EDTA, 0.1% SDS and 0.5% NP-40) containing a protease inhibitor cocktail. Five milligram of relevant GFP-tagged protein lysate was mixed with prewashed GFP beads and incubated overnight at 4 °C. Beads were subsequently washed on ice three times with washing buffer. For elution, the washed beads are resuspended in 100 μl of 2 × LDS sample buffer (Thermo Scientific) and boiled at 95 °C for 5 min. The eluate was loaded onto SDS page gel followed by western blot analysis using standard protocols. For western blot analysis, the following primary antibodies were used: anti-GFP (sc-9996, Santa Cruz), anti-GFP (2956, Cell Signaling), streptavidin-HRP (3999, Cell Signaling), anti-Gapdh (ab8245, Abcam) and anti-actin (ab8224, Abcam). Anti-H3K56 acetylation (39281; Activemotif), anti-K-acetylation (9441, Cell Signaling), anti-K-succinylation (PTM-401, PTM Biolabs), anti-Porin (16G9E6BC4, Life technologies, Carlsbad, CA, USA) and HRP-coupled anti-mouse or anti-rabbit secondary antibodies were from Jackson Immunoresearch. All western blots presented in the main text have been included as uncropped scans in Supplementary Fig. 6.

### Enrichment of biotinylated peptides and proteins

Proteins were denatured in 8 M guanidine-HCl (Gnd-HCl) before reduction and alkylation^63^. In-solution digest was performed overnight at 25 °C using the endopeptidase Lys-C, and the resulting peptide mixture was added to prewashed Strep-Tactin sepharose (IBA). Beads were washed three times in total with biotin buffer A (1 M Gnd-HCl, 10 mM HEPES pH 8.0 and 40 mM NH_4_HCO_3_) and twice with biotin buffer B (1 M Gnd-HCl, 10 mM HEPES pH 8.0 and 500 mM NaCl). Peptides were eluted twice with 8 M Gnd-HCl, pH 1.5 at 65 °C for 15 min and the combined eluate was diluted with 0.5% acetic acid and 0.1% trifluoroacetic acid (TFA) before purification^64^. Pull-down on biotinylated proteins was done as described above, but omitting the digest step.

### Immunofluorescence microscopy

Cells were fixed in LILLYs solution (Ampliqon, Copenhagen, Denmark) for 30 min at 30 °C, washed twice in PBS and sonicated briefly before zymolase treatment (Zymed, San Francisco, CA, USA). For GFP staining we used anti-GFP antibody (Santa Cruz Biotechnology, Santa Cruz, CA, USA), and for detection we used Alexa 488-conjugated goat-anti-mouse-antibody (Life Technologies). For biotin detection we used Alexa 549-conjugated streptavidin (Life Technologies). Microscopy was performed on a Zeiss LSM 780 confocal microscope (Carl Zeiss AG, Oberkuchen, Germany). For the Hst4–GFP localization, yeast cells were prepared for fluorescence microscopy^65^, and fluorophores were visualized on a Deltavision Elite microscope (Applied Precision, Inc.) equipped with a × 100 objective lens (Olympus U-PLAN S-APO, numerical aperture 1.4), a cooled Evolve 512 EMCCD camera (Photometrics, Japan) and a Insight solid-state illumination source (Applied Precision, Inc.). Pictures were processed with Volocity software (PerkinElmer). Images were acquired using softWoRx (Applied Precision, Inc.) software.

### Mitochondrial fractionation enrichment

Mitochondrial fractionation was essentially done as previously described^29^. Briefly, yeast cells were harvested by centrifugation, washed in distilled water and incubated for 10 min at 30 °C in pretreatment buffer (100 mM Tris-Cl pH 9.4, 50 mM dithiothreitol and 5 mM EDTA, pH 9.0). The cells were then washed in 1.2 M sorbitol before being resuspended at 0.15 g wet weight per ml of PM/sorbitol buffer (1.2 M sorbitol, 6.25 mM K_2_HPO_4_, 13.75 mM KH_2_PO_4_ and 1 mM MgCl_2_). Zymolyase 5,000 was added at 5 mg g^−1^ of cell wet weight and incubated for 50 min at 30 °C. Spheroplasts were harvested by centrifugation, washed in 1.2 M sorbitiol before dilution with one volume of buffer A (0.6 M mannitol, 10 mM Tris-Cl pH 7.4 and 0.1% BSA) and homogenization using a Douncer. Mitochondrial fractions were separated from cell debris by centrifugation and resuspended in buffer B (0.6 M mannitol and 10 mM Tris-Cl pH 7.4).

### Nicotinamide assay

Yeast strains grown in the absence or presence of biotin were harvested and resuspended 10 mM NaHPO_4_ containing protease inhibitor cocktail mix (Roche). Redissolved cells were dropped into liquid nitrogen and proteins were extracted using mechanical grinding (MM400 Ball Mill, Retsch). Cellular debris was removed by centrifugation, and protamine sulphate was added to 2 mg ml^−1^ final concentration. The supernatant was used for protein measurement and 20 μg from each cellular state was used for [^14^C]-nicotinamide conversion through the NAD^+^ salvage pathway^38^.

### NAD^+^/NADH measurements

WT and *hst4*▵ yeast strains grown in the absence or presence of biotin were harvested, washed twice in PBS and used in the fluorescent NAD/NADH detection kit (Cell Technology) according to the manufacturer's protocol. In short, pre-extraction NAD or NADH buffer was added to the cells and the cells were lysed by combining lysis buffer with short rounds of sonication. The lysates were heated to 60 °C for 15 min and neutralization NAD^+^ or NADH buffer was added together with reaction buffer. Fluorescent measurements were performed on a VarioSkan Flash (Thermo Scientific) on dilution series of the yeast strains in triplicates after a 90-min reaction time. Excitation was set to 550 nm and emission was measured at 595 nm.

### Acetyl-CoA measurements

Metabolite extractions were carried out according to the standard protocol^66^. In short, 1 ml of OD_600_=0.5 WT or *hst4*▵ yeast cultures grown in the absence or presence of biotin, were briefly quenched in 4 ml of 60% methanol/10 mM tricine at −40 °C. After a washing step, the cells were resuspended in 1 ml of 75% ethanol/0.5 mM tricine and incubated at 80 °C for 3 min. Cell debris were removed by two rounds of centrifugation. Samples were dried down in a speed vacuum and stored at −80 °C. Acetyl-CoA levels from yeast strains were measured by LC MS^27^. Samples were reconstituted in 10 mM ammonium formate and loaded onto an analytical column packed in-house with 3 μm C18 beads. Elution of acetyl-CoA was performed at a flow rate of 750 nl min^−1^ with a 10 min gradient of 0–22.5% acetonitrile in 10 mM ammonium formate. Acquisition of acetyl-CoA data was conducted on a Q Exactive Plus MS (Thermo Fisher Scientific) in targeted MS/MS mode^67^ using the transition specific for Acetyl-CoA^27^.

### Oxygen consumption measurements

All cells were grown in absence or presence of 2 μg l^−1^ biotin in TPP tubespin bioreactor tubes (Sigma-Aldrich) to allow free gas exchange. The cells were grown to mid-log phase before being resuspended to OD_600_=0.3 and collected by centrifugation. Oxygen consumption was determined using a XF96 Seahorse instrument (Seahorse Biosciences). After the initial calibration, a wait period of 5 min was allowed. Two-minute mix, 2-min measure cycles were repeated four times for the basal respiration before uncoupling oxidative phosphorylation using the ionophore FCCP (carbonyl cyanide 4-(trifluoromethoxy) phenylhydrazone) (Sigma-Aldrich) at 3 μM as final concentration. We used 12 wells for each strain condition. Seahorse oxygen consumption rate were repeated twice and results were further validated in cell suspension cultures using high-resolution respirometry (Oxygraph-2K, Oroboros instruments).

### Reactive oxygen measurements

ROS was measured by adding 10 μg of 2′,7′-dichlorofluorescein diacetate (Sigma-Aldrich) to 10^7^ cells and incubated at 30 °C for 2 h. Cells were washed with 1 ml of distilled water and resuspended in 0.1 ml PBS pH 7.3. Cells were observed in a light microscope (Axioskop 2 plus, Carl Zeiss, Göttingen, Germany) using the FITC HYQ filter. For each condition, four samples of around 100–300 cells were examined. The ratio of fluorescent to non-fluorescent cells was determined for each condition, and an unpaired *t*-test for differences between the mean was performed.

### Enrichment of acetylated peptides

Acetylated peptides were immunoenriched using the acetyl lysine antibody (ICP0388, Immunechem) at 4 °C^28^. Briefly, proteins were first digested with endoproteinase Lys-C (1 μg Lys-C per 100 μg protein) for 12–16 h at 25 °C. Lys-C activity was stopped by addition of TFA to a final concentration of 1% and the peptide solution was incubated at 4 °C for 1–2 h. The peptide mixture was then incubated with acetyl lysine antibody (ICP0388, Immunechem) at 4 °C, and immunoenriched peptides were eluted from anti-acetyl lysine antibody resin using acidified H_2_O (0.2% TFA). Peptide eluates were loaded directly onto a strong cation-exchange (SCX) microtip column prepared for chromatographic separation^68^. Peptide eluates were briefly evaporated to remove acetonitrile and then loaded onto C18 StageTips as described^68^ and subsequently analysed by quantitative MS.

### Mass spectrometric analysis

All MS experiments were performed on a nanoscale HPLC system (EASY-nLC from Proxeon Biosystems) connected to an Orbitrap Q-Exactive equipped with a nanoelectrospray source (Thermo Fisher Scientific). Each peptide sample was autosampled and separated on a 15-cm analytical column (75 μm inner diameter) in-house packed with 1.9-μm C18 beads (Reprosil Pur-AQ, Dr Maisch) with either a 1-h or 2-h gradient ranging from 5 to 40% acetonitrile in 0.5% acetic acid. The effluent from the HPLC was directly electrosprayed into the mass spectrometer. The Q-Exactive mass spectrometer was operated in a data-dependent acquisition mode, and all samples were analysed using previously described ‘fast' and ‘sensitive' acquisition methods^69^.

### Identification of peptides and proteins by MaxQuant

All raw data analysis was performed with MaxQuant software suite (www.maxquant.org)^70^ version 1.2.6.20 supported by the Andromeda search engine (www.andromeda-search.org). Data were searched against a concatenated target/decoy (forward and reversed) version of the yeast database containing 6,717 protein entries. Mass tolerance for searches was set to maximum 7 p.p.m. for peptide masses and 20 p.p.m. for HCD fragment ion masses. Data were searched with carbamidomethylation as a fixed modification and protein N-terminal acetylation, methionine oxidation and when required biotin or lysine acetylation as variable modifications. A maximum of two miscleavages was allowed while we required strict Lys-C specificity. Peptide assignments were statistically evaluated in a Bayesian model on the basis of sequence length and Andromeda score. We accepted only peptides and proteins with a false discovery rate of <1%, estimated on the basis of the number of accepted reverse hits. Protein sequences of common contaminants such as human keratins and proteases used were added to the database.

### Data analysis

GO annotation analyses were performed using the DAVID Functional Annotation Bioinformatics Microarray Analysis tool (http://david.abcc.ncifcrf.gov). *P* values were calculated separately for each GO term using Fisher's exact test, and only *P* values << 0.01 were considered statistically significant. For calculation of acetylation site overlap, only sites reported with a SILAC ratio and a localization score above 0.98 was used for the analysis. Since the light-SILAC state is identical between the investigated acetylomes, only a reported SILAC ratio signifies that the identified acetylation site is present in the light-SILAC experiments.

## Additional information

**How to cite this article:** Madsen, C. T. *et al.* Biotin starvation causes mitochondrial protein hyperacetylation and partial rescue by the SIRT3-like deacetylase Hst4p. *Nat. Commun.* 6:7726 doi: 10.1038/ncomms8726 (2015).

## Supplementary Material

Supplementary Figures Supplementary Figures 1-6

Supplementary Data 1 List of identified biotinylated proteins and corresponding modified lysine residues

Supplementary Data 2 List of proteome abundance changes upon biotin starvation (Experiment 1) and biotin supplement (Experiment 2) in yeast cells

Supplementary Data 3 List of lysine acetylation abundance changes upon biotin starvation (Experiment 1) and biotin supplement (Experiment 2) in yeast cells

Supplementary Data 4 List of lysine acetylation abundance changes upon genetic deletion of various yeast sirtuins (Sir2p, Hst2p and Hst4p)

Supplementary Data 5 List of primers used for generation of Sec28p K27R mutant
