# Supplementary material for: Biotin starvation causes mitochondrial protein hyperacetylation and partial rescue by the SIRT3-like deacetylase Hst4p
Source: Nat Commun. 2015 Jul 9;6:7726. doi: 10.1038/ncomms8726 (PMC4510963; doi:10.1038/ncomms8726)
Supplement: Supplementary Figures — 1-6 [file ncomms8726-s1.pdf]

Supplementary Figure 1

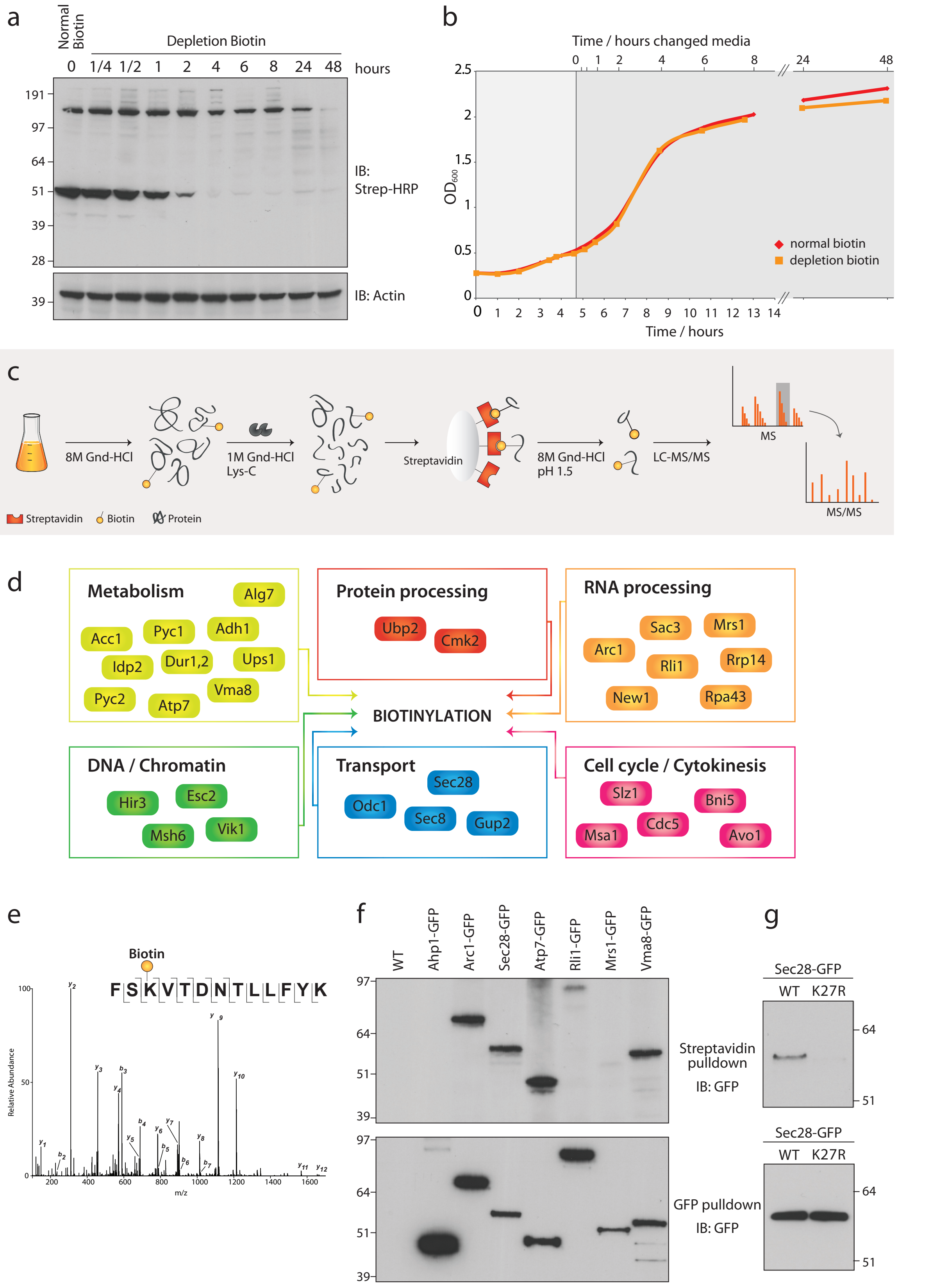

Supplementary Figure 1: Identification of novel biotinylated proteins by high stringency peptide enrichment procedure. (A) Time course evaluation of biotin depletion. Yeast grown with normal amounts of biotin (2 ug/l) were harvested at OD600 = 0.5, washed extensively in sterile H2O and redissolved in depletion media. State of biotinylation at the indicated time points after change to depletion media was determined by WB analysis using streptavidin-HRP and actin antibody. (B) Yeast grown with normal amounts of biotin (2 ug/l) were harvested at OD600 = 0.5, washed extensively in sterile H2O and redissolved in media containing 2 ug/l biotin (red line) or in depletion media (orange line). (C) Enrichment strategy for identification of biotinylated proteins. Exposed biotinylated peptides are enriched on a streptavidin column and eluted off with 8 M Gnd-HCl at pH 1.5 and analyzed by LC-MS. (D) Distribution of identified biotinylated proteins in cellular pathways. (E) The Sec28p MS/MS peptide fragment spectra identify lysine 27 (K27) as the site of biotin attachment. (F) Validation of novel biotinylation targets. Protein extract from indicated GFP tagged strains were used for streptavidin pulldown and GFP WB (upper panel) or GFP pulldown to ensure the presence of the GFP-tagged protein (lower panel). WT and Ahp1-GFP lysate function as negative controls and Arc1-GFP as positive control. (G) K27R mutation abolishes Sec28p biotinylation, whereas the WT Sec28p is readily enriched on streptavidin beads (upper panel). Control for expression (lower panel).

Supplementary Figure 2

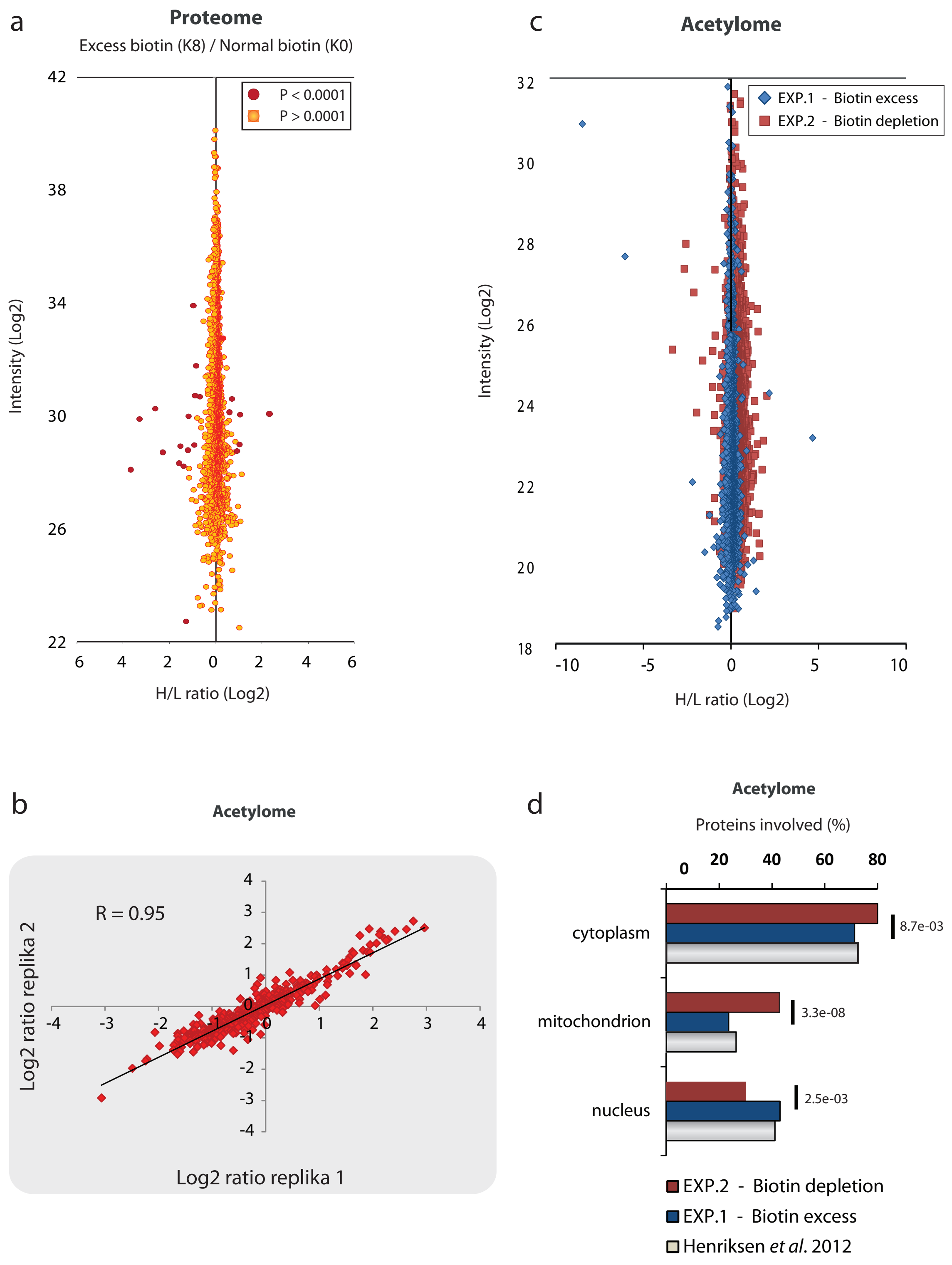

Supplementayr Figure 2: Proteome and acetylome changes in response to biotin availability.
(A) Proteome changes in EXP. 1 (biotin excess). The intensities of the identified proteins are plotted against the corresponding normalized heavy/light ratio. Significant outliers (red color) are determined using a Benjamini-Hochberg test with a FDR of $p < 0.0001$ from the Perseus software as part of Maxquant. (B) Ratio plots and Pearson correlation for acetylated proteins identified from two replica EXP.2 (biotin depletion) experiments. (C) Distribution of acetylated peptides identified in the excess EXP.1 (blue) and depletion EXP.2 (red) biotin experiment. The intensities are plotted against the corresponding normalized heavy/light ratio. The excess experiment exhibits a narrow distribution indicating very little regulated acetylation changes takes place. In contrast the depletion experiment reveals a much broader and shifted distribution indicative of substantial acetylation change. (D) Cellular distribution of acetylated proteins in EXP.1 (biotin excess), EXP.2 (biotin depletion) and from an independent study in S. cerevisiae (Henriksen et al., 2012). The excess biotin experiment has a distribution which follows closely the previous published data set, whereas the depletion biotin experiment is overrepresented in the mitochondria. P-values were determined using a Benjamini-Hochberg correction for multiple hypothesis testing.

Supplementary Figure 3

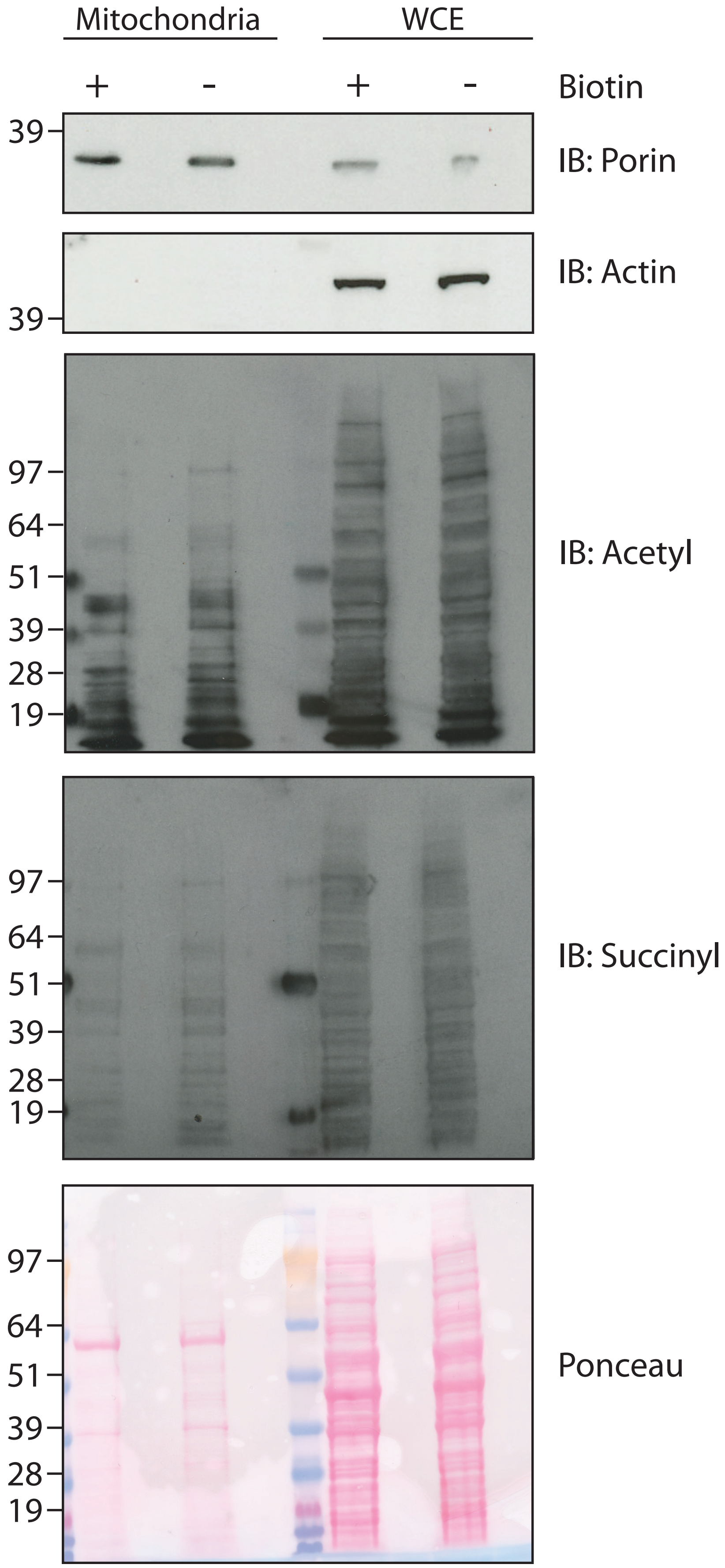

Supplementary Figure 3: Mitochondrial and whole cell extract (WCE) lysine acetylation and lysine succinylation western bl
Including control blot for the efficiency of the mitochondrial fraction: Porin specific antibody and actin specific antibody. Additionally included Ponceau blot for evaluation of starting material for mitochondrial and WCE.

Supplementary Figure 4

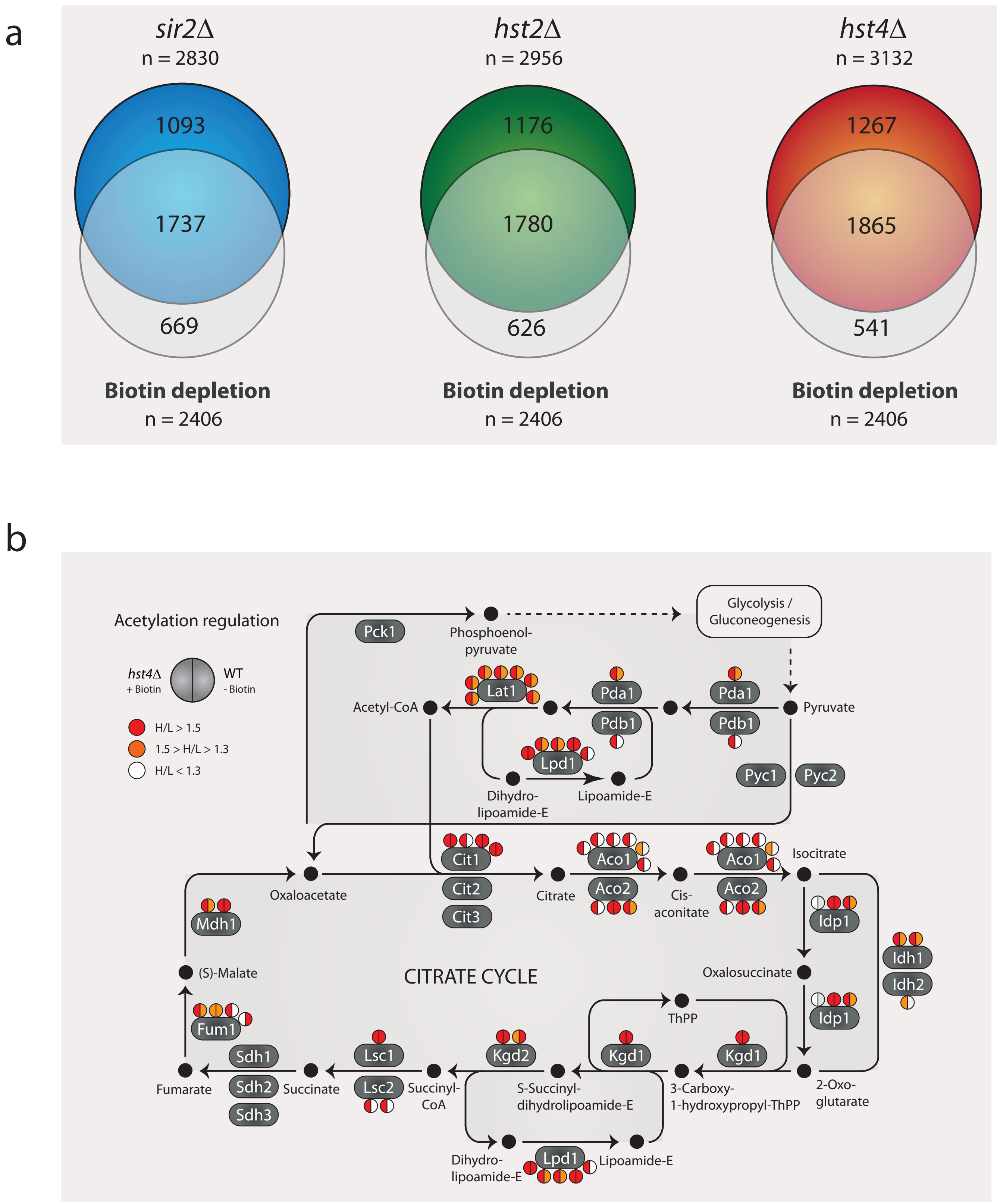

Supplementary Figure 4: Acetylation changes in WT lacking biotin overlaps with the hst4 mutant strain.
(A) Overlap between acetylation sites identified in biotin depletion (EXP.2) experiment and those identified in each sirtuin mutant. The large overlap signifies technical high reproducibility. (B) Overlap in acetylation response between WT without biotin and the hst4 mutant in the citrate cycle. Proteins with individual acetylated lysines represented as round circles. Left side of the circle is the level of regulation determined in the hst4 mutant and the right side the corresponding measured acetylation change in the WT grown without biotin.

Supplementary Figure 5

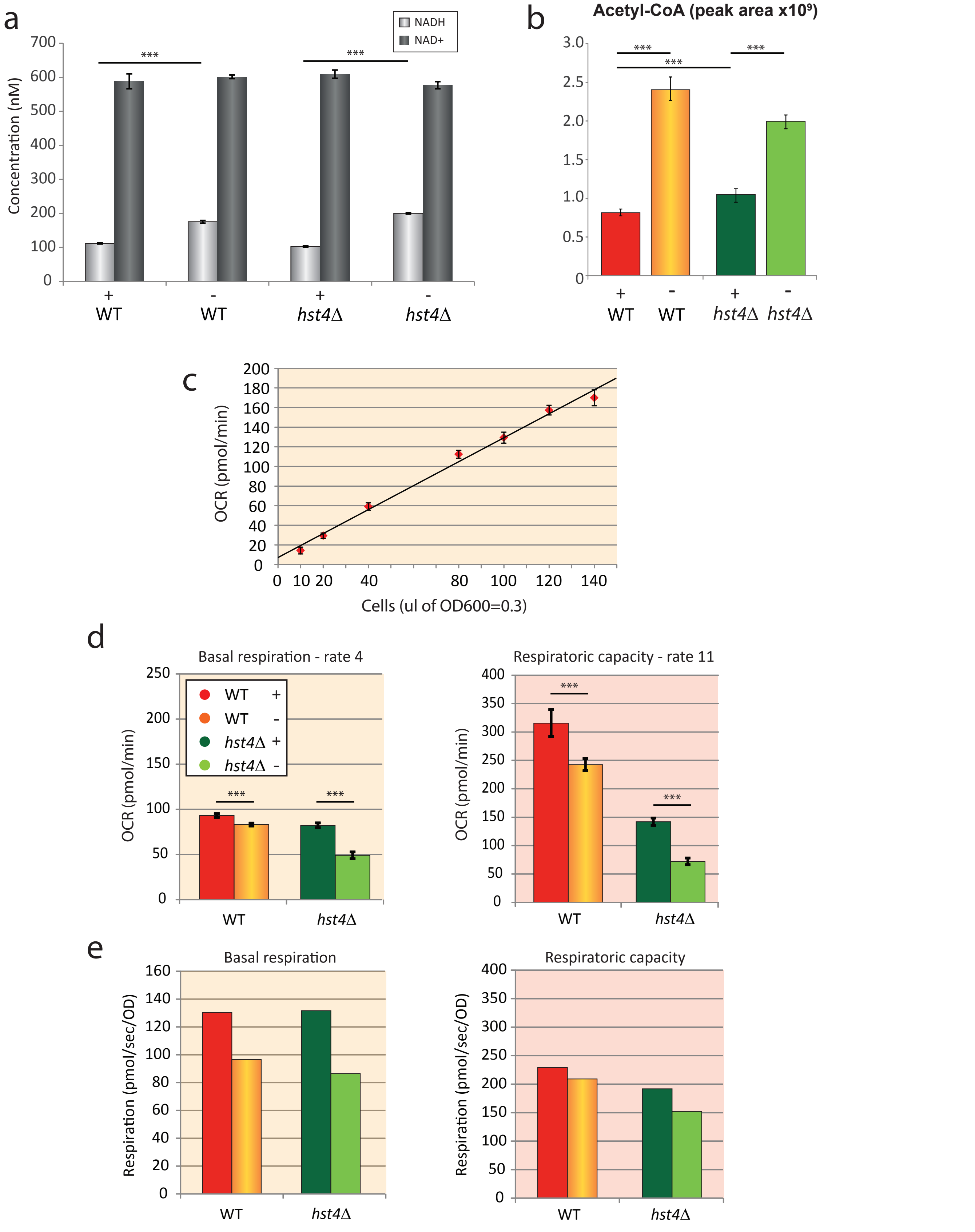

Supplementary Figure 5: Cells lacking biotin exhibit metabolic inflexibility.
(A) NAD+ and NADH concentration in WT and the hst4 mutant as quantified from standard curve measurements. Data shown is mean±SD from three independent replica experiments. (B) Relative acetyl-CoA levels from the four yeast strains as determined by LC-MS. Expressed percentages are in comparison to the WT supplemented with biotin, with error bars denoting the standard deviation of six replicates (biological triplicates measured in duplicate).
(C) Oxygen consumption rate (pmoles/min) is linear correlated with number of cells used in Seahorse instrument.
(D) OCR measurements in Seahorse instrument at rate 4 (Basal respiration) and at rate 11 (Maximum respiratory capacity) with 3 uM FCCP in WT and hst4 mutant. Data shown is mean ± standard deviation from 12 measurements for each condition. (E) Respiration measurements (pmol/sec/OD) in cell suspension cultures using an Oxygraph-2K (Oroboros instruments). . *** $p < 0.001$. For figures a), b) and d) an unpaired t-test was performed to test difference between the mean from three biological replica experiments.

Supplementary Figure 6

Figure 1b

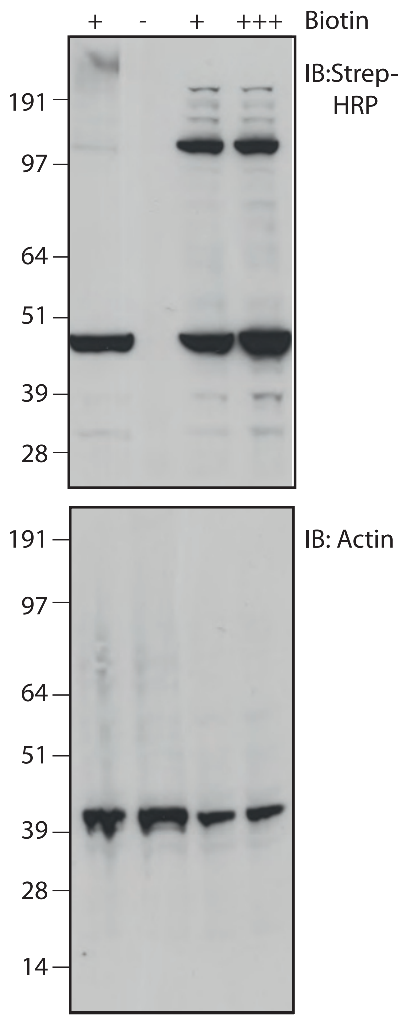

Figure 2b

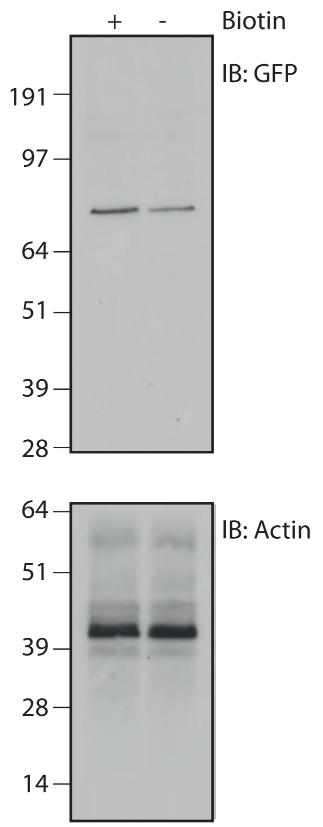

Figure 4b

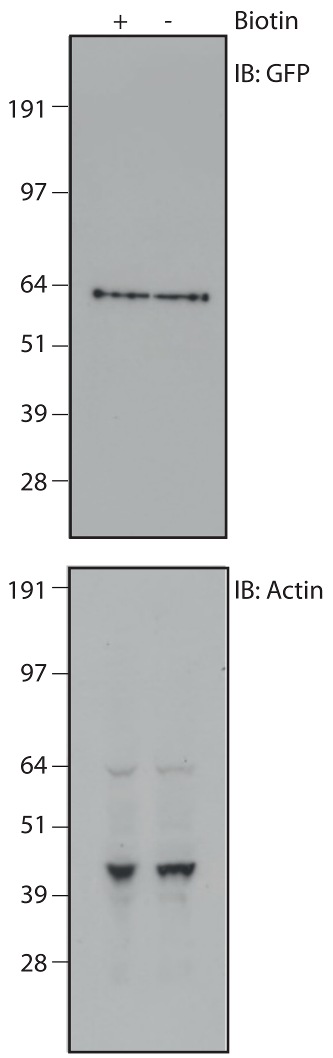

Figure 4c

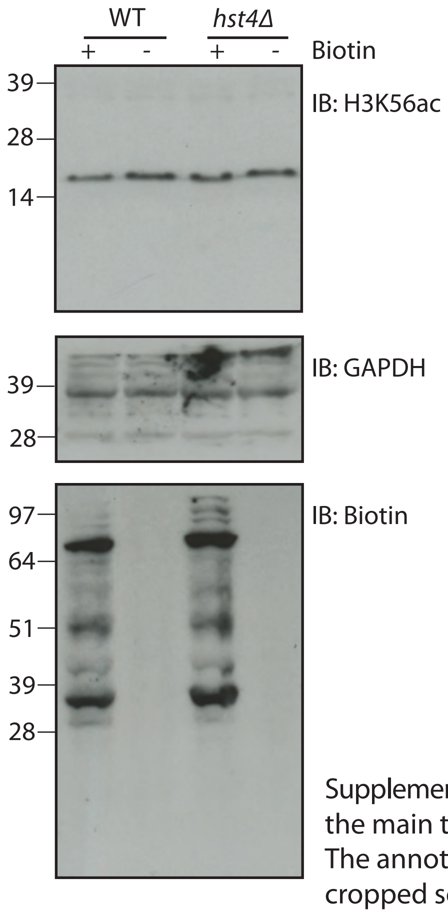

Figure 4d

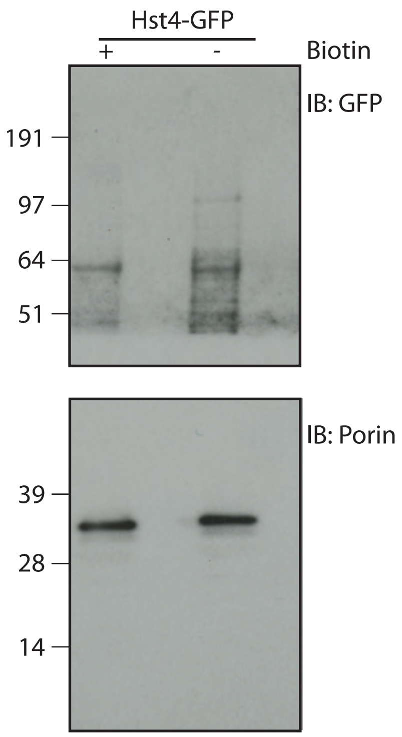

Supplementary Figure 6: Uncropped scans related to presented western blots in the main text.
The annotation of each scan refers to the main figure where the cropped scan (western blot) is presented
